# Evaluation of rapeseed varieties using novel integrated fuzzy PIPRECIA – Fuzzy MABAC model

**DOI:** 10.1371/journal.pone.0246857

**Published:** 2021-02-25

**Authors:** Miroslav Nedeljković, Adis Puška, Suzana Doljanica, Saša Virijević Jovanović, Pavle Brzaković, Željko Stević, Dragan Marinkovic

**Affiliations:** 1 Agricultural Faculty, University of Bijeljina, Bijeljina, Bosnia and Herzegovina; 2 Government of the Brčko District of BiH, Brčko, Bosnia and Herzegovina; 3 Faculty of Applied Management, Economics and Finance, University Business Academy, Novi Sad, Serbia; 4 Faculty of Transport and Traffic Engineering, University of East Sarajevo, Sarajevo, Bosnia and Herzegovina; 5 Faculty of Mechanical Engineering and Traffic Systems, TU Berlin, Berlin, Germany; Universita degli Studi del Molise, ITALY

## Abstract

Decision making is constantly present in agriculture. Choosing the wrong variety carries the risk that the investment in terms of sowing does not pay off at all. Therefore, it is necessary to choose the variety that gives the best results. In order to achieve this, it is necessary to apply multi-criteria decision-making of available varieties, which is, in this paper, done on the example of hybrid varieties of rapeseed that were created by selection at the Institute of Field and Vegetable Crops in Novi Sad. By applying fuzzy logic, a novel integrated Multi-Criteria Decision-Making (MCDM) model is developed and rapeseed varieties were evaluated. For determining four main and 20 subcriteria, fuzzy PIPRECIA (PIvot Pairwise RElative Criteria Importance Assessment) method has been applied based on fuzzy Bonferroni operator, while for ranking alternatives fuzzy MABAC (Multi-Attributive Border Approximation area Comparison) method has been used. The results obtained using the novel integrated fuzzy MCDM model showed that the variety A2 – Zorica has the best results, followed by A1 - NS Ras, while the worst results were seen by the variety A5 - Zlatna. These results were confirmed using other five fuzzy MCDM methods. Sensitivity analysis—changing criteria weights showed the worst results in the variety A6 - Jovana, which took last place in the application of 18 scenarios. The presented model and the results of this research will help farmers to solve this decision problem.

## 1. Introduction

Rapeseed is one of the main sources of vegetable oil in the world. In addition to the relatively high and stable price, which is probably a decisive factor in the decision of producers to sow rapeseed on their fields [1], rapeseed has a number of characteristics that enhance its production [2]. It is used to produce the oil of excellent quality for human consumption and the processing industry [3]. Furthermore, it is also used to obtain protein components for domestic animal nutrition [4]. Rapeseed is also recommended in organic agriculture and as a component in joint crops [5, 6].

According to Food and Agriculture Organization (FAO) data, the area of rapeseed reached 39,579,575 ha, and its production 75,001,457 tons in the world in 2018. The production of this oilseed in the European Union (EU) (28) reached the value of 19,974,567 tons in the same year, which immediately put it behind its largest producer, which is Canada (20,342,600 tons). When it comes to the production of this oilseed in Bosnia and Herzegovina, it can be seen that it is very modest and, according to the same source, it amounted to 26,775 tons in 2018, which is only 0.13% of production in the EU (28) or 0.03% in the world.

Considering the growing importance of this industrial plant and the growing production potential it has, many agricultural farms and agro-industrial complexes in Bosnia and Herzegovina should make decision to produce it. The main role in the decision to sow this oilseed is played by the selection and evaluation of the current variety (hybrid) that is available on the market. Rapeseed hybrid selection is necessary to improve production. Constant improvement of existing hybrids and introduction of new ones is needed. In order to achieve this, it is essential to choose the best hybrid and improve it. However, the main problem is the existence of a large number of hybrids. Reducing the number of them is achieved by comparing the existing hybrids. Each of the hybrids should be evaluated and compared to the existing ones in order to determine which of them has the best characteristics.

The aim of this paper is to develop a novel integrated fuzzy MCDM model of support for deciding on potential sowing with some of the analyzed rapeseed hybrids. The realization of this aim will result in the selection of the best hybrid that will serve to further improvement of the rapeseed production. It is necessary to choose the hybrid that shows the best results since it will serve as a basis for creating new rapeseed hybrids. For the purposes of the research, the selection and analysis refer to six varieties of rapeseed hybrids created by selection at the Institute of Field and Vegetable Crops in Novi Sad. The fuzzy MCDM methodology is used to decide on several alternatives, and the evaluation is performed according to criteria anonymously and differently set by several experts in the field. The contribution of this paper is in the following:

- Forming models that will, through the selection of the best hybrid, serve for further improvement of rapeseed hybrids;
- Application of a new methodology based on fuzzy logic;
- Introduction of new analysis for confirming research results.

*Fuzzy* logic allows the introduction of a mean value defined between the traditional attitudes of yes/no, true/false, black/white, and so on [7]. The use of the fuzzy approach is based on linguistic values provided by the experts. The concept of linguistic values is useful in dealing with situations that are too complex or not well defined to be valued in quantitative terms. Linguistic values are closer to human thinking and are easier to use when evaluating quantitative indicators.

In addition to the introduction, this paper is divided into six other sections. In the second section, a review of the literature on previous research in agriculture using MCDM methods was performed. This was followed by an explanation of the basics of fuzzy logic, fuzzy Bonferroni mean (BM) operator, fuzzy PIPRECIA and the MABAC method presented in the third section. In the fourth section of this paper, the case study was presented. The fifth section was in charge of the results of this research. In the sixth section, sensitivity analysis was applied to confirm the obtained research results. The seventh section was in charge of giving the most important conclusions of this research.

## 2. Literature review

Decision-making in agriculture is an unavoidable phase in business. It is a complex business since decisions due to the impossibility of quantification are often made on the basis of qualitative data or, even more often, in combination with existing quantitative data [8, 9]. The problem becomes more complicated with the fact that unforeseen climatic conditions and numerous risks, caused by political, economic and social phenomena in the society, are increasingly present. In addition, different varieties and types of fruit and vegetables are available to farmers, and they need to decide what to plant and in what way. In order to make a decision in agriculture, it is necessary to consider certain alternatives that are available with certain criteria. Due to the existence of several criteria that need to be considered when making a decision, multi-criteria decision analysis (MCDA) methods are used.

Deepa et al. [10] used the methods VIKOR (Multicriteria Optimization and Compromise Solution), TOPSIS (Technique for Order Preference by Similarity to an Ideal Solution), entropy, and standard deviation (VTOPES) for ranking sustainable farms for sugar cane production. Dooley et al. [11] discussed MCDA methods and their application in agriculture on the example of three case studies and provided guidelines on how these methods can be used in agriculture. Crnčan et al. [12] used the AHP (Analytic Hierarchy Process) method in the selection of egg production systems based on the assessment of the respondents. Latinopoulos [13] used the basics of the MCDA to develop a MOP (Mathematical Optimization Programming) model that served to efficiently allocate water and land resources in rural Greece. Rozman et al. [14] used the DEX method (Decision EXpert) to evaluate dairy farms in Bosnia and Herzegovina. Bogdanović and Hadžić [15] performed a strategic analysis of investments in agriculture using the AHP method and found that perennial plantations are a better long-term investment strategy than conventional crop production.

Pozderec et al. [16] used the DEX and AHP methods to integrate the developed specific technological-economic simulation models for vegetable production into a greenhouse in integrated and organic production. Bartzas and Komnitsas [17] combined life cycle analysis, environmental risk assessment together with the AHP method to select the most sustainable agricultural management practices at the regional level. Yazdani et al. [18] used the extended Step-wise Weight Assessment Ratio Analysis method combining gas and multicriteria analysis to manage the circular economy in agriculture. Nikoloski et al. [19] used the DEX method to create a model of decision support on farm relocation in horticulture based on criteria regarding region and specific farm. Elleuch et al. [20] used Fuzzy Multi-Criteria Decision-Making (FMCDM) methods and MOP for the water distribution problem.

Based on this brief review of the literature, it could be seen that different MCDA methods were used and different problems in agriculture were solved. Jayakumar and Hari Ganesh [21] used the fuzzy multicriteria group decision making (FMCGDM) model for rice variety selection. Draginčić et al. [22] used the Simple Additive Weighting (SAW) method, the AHP and Cardinal consensus model using the Group multi-criteria decision making (GMCDM) when selecting a grape variety. Rozman et al. [23] used the DEX method to select the plum variety that gives the best results in integrated fruit production Maksimović et al. [24] used the DEX method to select the apple variety that gives the best results in North-western Bosnia and Herzegovina. Also, Maksimović et al. [25] used the DEX method to select the fruit species that give the best results in integral fruit production. Milovanović and Stojanović [26] used the AHP method to select cherry varieties. Rozman et al. [27] used the AHP method when evaluating new apple cultivars. Paunović et al. [28] used the Fuzzy Interference System (FIS) to evaluate cherry varieties for orchard raising purposes. Based on a brief review of the literature, it can be concluded that MCDA methods can be used to evaluate different varieties for sowing purposes.

## 3. Methods

*Fuzzy* logic is used to model inaccurate information derived from human thinking [29]. In order to make a decision, human subjectivity should be taken into account, and not only the objective probabilities of the occurrence of an event, because it is not possible to have complete information. When making decisions to deal with very complex problems, one does not have to use rigidity to specify the description and thinking about phenomena as much as possible, but one can go in the opposite direction and allow the descriptions to be imprecise in the spirit of natural language [30].

## 3.1. Operations on fuzzy numbers

A fuzzy number $\bar{A}$ on R to be a triangular fuzzy number (TFN) if its membership function $\mu_{\bar{A}}(x)$: R→[0,1] is equal to following Eq (1):

$$\mu_{\bar{A}}(x)=\left\{ \begin{array}{l} \frac{x-l}{m-l}\;l\leq x\leq m\\ \frac{u-x}{u-m}\;m\leq x\leq u\\ 0\;otherwise \end{array}\right.$$ (1)

From Eq (1), *l* and *u* mean the lower and upper bounds of the fuzzy number $\bar{A}$, and *m* is the modal value for $\bar{A}$. The TFN can be denoted by $\bar{A}=(l,m,u)$.

The operational laws of TFN $\bar{A}=(l_{1},m_{1},u_{1})$ and $\bar{A}=(l_{2},m_{2},u_{2})$ are displayed as following equations [31–33].

Addition:

$$\bar{A_{1}}+\bar{A_{2}}=(l_{1},m_{1},u_{1})+(l_{2},m_{2},u_{2})=(l_{1}+l_{2},m_{1}+m_{2},u_{1}+u_{2})$$ (2)

Multiplication:

$$\bar{A_{1}}\times \bar{A_{2}}=(l_{1},m_{1},u_{1})\times (l_{2},m_{2},u_{2})=(l_{1}\times l_{2},m_{1}\times m_{2},u_{1}\times u_{2})$$ (3)

Subtraction:

$$\bar{A_{1}}-\bar{A_{2}}=(l_{1},m_{1},u_{1})-(l_{2},m_{2},u_{2})=(l_{1}-u_{2},m_{1}-m_{2},u_{1}-l_{2})$$ (4)

Division:

$$\frac{\bar{A_{1}}}{\bar{A_{2}}}=\frac{\left(l_{1},m_{1},u_{1}\right)}{\left(l_{2},m_{2},u_{2}\right)}=\left(\frac{l_{1}}{u_{2}},\frac{m_{1}}{m_{2}},\frac{u_{1}}{l_{2}}\right)$$ (5)

Reciprocal:

$${\bar{A_{1}}}^{-1}={\left(l_{1},m_{1},u_{1}\right)}^{-1}=\left(\frac{1}{u_{1}},\frac{1}{m_{1}},\frac{1}{l_{1}}\right)$$ (6)

### 3.2. Fuzzy Bonferroni mean (BM) operator

Surveys were conducted anonymously to collect the data necessary for this research from five experts in the field. Since five experts participate in the research, the values of the interval fuzzy vector of weight coefficients are aggregated using a fuzzy Bonferroni aggregator [34], Eq (7). The Bonferroni mean (BM) operator [35] was used to aggregate the vector of weight coefficients because it allows the representation of the interrelationships between the aggregation elements [36].

$${\tilde{a}}_{ij}=(a_{ij}^{l},a_{ij}^{m},a_{ij}^{u})=\left\{ \begin{array}{l} a_{ij}^{l}={\left(\frac{1}{e(e-1)}\sum_{\begin{array}{l} i,j=1\\ i\neq j \end{array}}^{e}a_{i}^{l}{}^{p}⊗a_{j}^{l}{}^{q}\right)}^{\frac{1}{p+q}}\\ a_{ij}^{m}={\left(\frac{1}{e(e-1)}\sum_{\begin{array}{l} i,j=1\\ i\neq j \end{array}}^{e}a_{i}^{m}{}^{p}⊗a_{j}^{m}{}^{q}\right)}^{\frac{1}{p+q}}\\ a_{ij}^{u}={\left(\frac{1}{e(e-1)}\sum_{\begin{array}{l} i,j=1\\ i\neq j \end{array}}^{e}a_{i}^{u}{}^{p}⊗a_{j}^{u}{}^{q}\right)}^{\frac{1}{p+q}} \end{array}\right.$$ (7)

where *e* represents the number of experts participating in the research, while p, q ≥ 0 are set of non-negative numbers.

### 3.3. Fuzzy PIPRECIA method

Steps of this method are the following [9, 37, 38]:

Step 1. Forming set of criteria and sorting the criteria according to marks from the first to the last, and this means that they need to be sorted unclassified.

Step 2. Each decision-maker individually evaluates pre-sorted criteria by starting from the second criterion.

$$\bar{s_{j}^{r}}=\left\{ \begin{array}{l} >\bar{1}\;if\;C_{j}>C_{j-1}\\ =\bar{1}\;if\;C_{j}=C_{j-1}\\ <\bar{1}\;if\;C_{j}<C_{j-1} \end{array}\right.$$ (8)

$\bar{s_{j}^{r}}$ denotes the assessment of criteria by a decision-maker *r*.

Step 3. Determining the coefficient $\bar{k_{j}^{}}$

$$\bar{k_{j}^{}}=\left\{ \begin{array}{l} =\bar{1}\;if\;j=1\\ 2-\bar{s_{j}^{}}\;if\;j>1 \end{array}\right.$$ (9)

Step 4. Determining the fuzzy weight $\bar{q_{j}^{}}$

$$\bar{q_{j}^{}}=\left\{ \begin{array}{l} =\bar{1}\;if\;j=1\\ \frac{\bar{q_{j-1}^{}}}{\bar{k_{j}}}\;if\;j>1 \end{array}\right.$$ (10)

Step 5. Determining the relative weight of the criterion $\bar{w_{j}^{}}$

$$\bar{w_{j}}=\frac{\bar{q_{j}}}{\sum_{j=1}^{n}\bar{q_{j}}}$$ (11)

In the following steps, the inverse methodology of fuzzy PIPRECIA method requires to be applied.

Step 6. Performing the assessment but this time starting from a penultimate criterion.

sjr¯'={>1¯ifCj>Cj+1=1¯ifCj=Cj+1<1¯ifCj<Cj+1 (12)

Step 7. Determining the coefficient kj¯'

kj¯'={=1¯ifj=n2−sj¯'ifj>n (13)

Step 8. Determining the fuzzy weight qj¯'

qj¯'={=1¯ifj=nqj+1'¯kj¯'ifj>n (14)

Step 9. Determining the relative weight of the criterion wj¯'

wj¯'=qj¯'∑j=1nqj¯' (15)

Step 10. In order to determine the final weights of criteria, it is necessary to perform the defuzzification of the fuzzy values $\bar{w_{j}^{}}$ and wj¯' first.

wj¯''=12(wj+wj') (16)

Step 11. Checking the results obtained by applying Spearman and Pearson correlation coefficients.

### 1.1. Fuzzy MABAC method

Multi-Attributive Border Approximation area Comparison (MABAC) developed by Pamučar and Ćirović [39] is one of the recent approaches to multi-attribute decision making (MCDM/MADM) problems. The basic assumption of the MABAC method is reflected in the definition of the distance of the alternative from the boundary approximate domain. The boundary approximate area represents the average value for all alternatives. If the alternative is above that value, its value will be positive and vice versa.

The application of the fuzzy MABAC method is done by using 7 steps [40].

Step 1. Forming the initial decision matrix. In the first step the evaluation of *m* alternatives by *n* criteria is performed.

Step 2. Normalization of the initial matrix elements. The elements of the normalized matrix are obtained by using the expressions:

For benefit-type criteria:

$$\tilde{t}=(t_{ij}^{l},t_{ij}^{m},t_{ij}^{u})=\left(\frac{x_{id}^{l}}{x_{ij}^{u}},\frac{x_{id}^{l}}{x_{ij}^{m}},\frac{x_{id}^{l}}{x_{ij}^{l}}\right)\;if\;j\in C$$ (17)

For cost-type criteria:

$$\tilde{t}=(t_{ij}^{l},t_{ij}^{m},t_{ij}^{u})=\left(\frac{x_{ij}^{l}}{x_{id}^{u}},\frac{x_{ij}^{m}}{x_{id}^{u}},\frac{x_{ij}^{u}}{x_{id}^{u}}\right)\;if\;j\in B$$ (18)

where *l* is the first *fuzzy* number, *m* is the second *fuzzy* number and *u* is the third *fuzzy* number.

Step 3. Calculation of the weighted matrix (V) elements [41].

$${\tilde{v}}_{ij}=w_{i}∙{\tilde{t}}_{ij}+w_{i}$$ (19)

where *w*_*i*_ represents the weighted coefficients of the criterion.

Step 4. Determination of the approximate border area matrix (G).

$$g={\left(\prod_{j=1}^{m}{\tilde{v}}_{ij}\right)}^{1/m}$$ (20)

where m represents total number of alternatives.

Step 5. Calculation of the matrix elements of alternatives distance from the border approximate area. The distance of the alternatives from the border approximate area (${\tilde{q}}_{ij}$) is defined as the difference between the weighted matrix elements (V) and the values of the border approximate areas (G).

$$\tilde{Q}=\tilde{V}-\tilde{G}$$ (21)

Now, boarder approximation area value for each criteria function serves as reference point/benchmark value for criteria-wise performance of an alternative *A*_*i*_. Each individual candidate will belong to the three different areas namely the border approximation area (*G*), upper approximation area (*G*^+^), and lower approximation area (*G*^−^). The ideal alternative ($A_{i}^{+}$) can be found in the upper approximation area (*G*^+^) whereas the lower approximation area (*G*^−^) contains the anti-ideal alternative ($A_{i}^{-}$) [42].

$${\tilde{A}}_{i}\in \left\{ \begin{array}{c} {\tilde{G}}^{+}\;if\;{\tilde{q}}_{ij}>0\\ \tilde{G}\;if\;{\tilde{q}}_{ij}=0\\ {\tilde{G}}^{-}\;if\;{\tilde{q}}_{ij}<0 \end{array}\right.$$ (22)

In order for the alternative *A*_*i*_ to be chosen as the best from the set, it is necessary for it to belong, by as many as possible criteria, to the upper approximate area (${\tilde{G}}^{+}$) [43]. The higher value ${\tilde{q}}_{i}\in {\tilde{G}}^{+}$ indicates that the alternative is closer to the ideal alternative, while the lower value q${\tilde{q}}_{i}\in {\tilde{G}}^{-}$ indicates that the alternative is closer to the anti-ideal alternative [44].

Step 6. Ranking of alternatives. The calculation of the values of the criteria functions by alternatives is obtained as the sum of the distance of alternatives from the border approximate areas (${\tilde{q}}_{i}$). By summing up the matrix $\tilde{Q}$ elements per rows, the final values of the criteria function of alternatives are obtained

$${\tilde{S}}_{i}=\sum_{j=1}^{n}{\tilde{q}}_{ij},\;j=1,2,\ldots ,n,i=1,2,\ldots ,m.$$ (23)

Step 7. Final ranking of alternatives. By defuzzification of the obtained values ${\tilde{S}}_{i}$, the final rank of alternatives is obtained.

$$S=\frac{t_{1}+4t_{2}+t_{3}}{6}$$ (24)

The best alternative is the one that has the greatest value [45].

## 4. Case study

From the literature review it could be seen that there are different methods of MCDA that can be used in different problems in agriculture. In order to pursue the aim of the research in this paper, the evaluation of rapeseed varieties, originated at the Institute of Field and Vegetable Crops in Novi Sad, will be performed. Each of these varieties has its advantages and disadvantages, so this paper will develop a decision model that will serve as the basis for a detailed analysis of these varieties. By analysing rapeseed hybrids produced at the Institute of Field and Vegetable Crops in Novi Sad, the best hybrid that will serve as the basis for the creation of new hybrids, will be selected. Methods developed on the principles of fuzzy logic will be used. The following steps were used to collect data for the purposes of this study.

**Fig 1:**
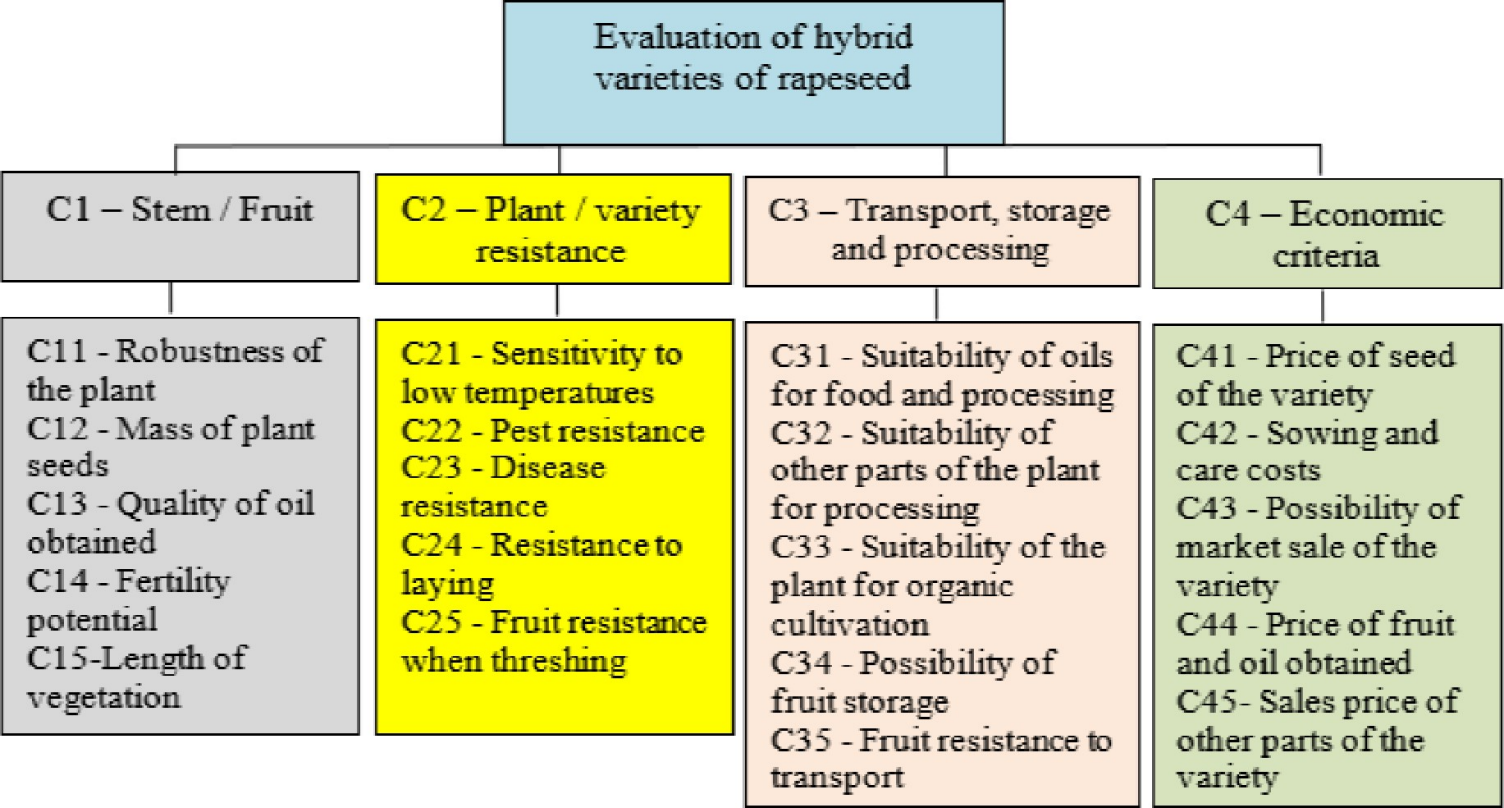
Decision making model.

**Step 1** Selection of criteria according to which these varieties of rapeseed will be evaluated. In this step, a review of previous research was performed first and certain criteria were selected. Anonymous surveys were organized to collect data from the experts who performed the evaluation. They selected several criteria that were grouped into four groups according to which the evaluation of rapeseed varieties would be performed. Based on these criteria, a decision-making model was created (Fig 1). The decision model has four main criteria: C1 stem or fruit, C2 resistance of the plant or variety, C3 transport, storage and processing of varieties, C4 economic criterion. All these criteria are further divided into five sub-criteria. The reason for this was that all the criteria would have the equal number of sub-criteria and so that none of them would stand out. Each of the criteria and sub-criteria will be evaluated by experts using linguistic values from Fuzzy PIPRECIA method.

**Step 2** Selecting alternatives to be evaluated. For the purposes of this research, the experts selected six different hybrid varieties of rapeseed: A1—NS Ras, A2—Zorica, A3—Anna, A4—Jasna, A5—Zlatna and A6—Jovana. Selected rapeseed varieties will be evaluated using linguistic values ranging from Very bad (VB) to Very good (VG) values. As with the evaluation of criteria, seven linguistic assessments are available for the evaluation of alternatives. By using linguistic assessments, decision-making approaches human thinking and is much easier.

**Step 3** Data collection and ranking of alternatives. After the experts have evaluated the criteria and alternatives, the assessments are collected and the initial decision matrix is formed, which is the basis for the implementation of the MABAC method. Prior to that, all criteria weights are calculated using fuzzy PIPRECIA method based on fuzzy BM operator. The steps of the fuzzy MABAC method are then applied and the ranking orders of the alternative are determined. To confirm these results, a sensitivity analysis will be performed where other results will be checked using other fuzzy methods. Furthermore, it will examine how sensitive the alternatives are to changes in the weights of the sub-criteria.

## 5. Research results

Before ranking hybrid varieties of rapeseed, it is necessary to calculate the weights of the criteria. The experts first evaluated the main criteria and then the sub-criteria for the individual main criteria. In Table 1 the evaluation of main criteria by all experts and their aggregation using fuzzy operator is presented.

**Table 1:** Expert assessments of the weight of the main criteria.

PIPR.	C1			C2			C3			C4		
E1				1.400	1.600	1.650	0.333	0.400	0.500	1.300	1.450	1.500
E2				1.300	1.450	1.500	0.400	0.500	0.667	1.300	1.450	1.500
E3				1.300	1.450	1.500	0.400	0.500	0.667	1.300	1.450	1.500
E4				1.200	1.300	1.350	0.500	0.667	1.000	1.100	1.150	1.200
E5				1.400	1.600	1.650	0.333	0.400	0.500	1.300	1.450	1.500
BM				1.319	1.479	1.529	0.392	0.491	0.660	1.259	1.389	1.439
PIPR-I	C4			C3			C2			C1		
E1				0.333	0.400	0.500	1.300	1.450	1.500	0.286	0.333	0.400
E2				0.333	0.400	0.500	1.200	1.300	1.350	0.333	0.400	0.500
E3				0.333	0.400	0.500	1.200	1.300	1.350	0.333	0.400	0.500
E4				0.500	0.667	1.000	1.100	1.150	1.200	0.400	0.500	0.667
E5				0.333	0.400	0.500	1.300	1.450	1.500	0.286	0.333	0.400
BM				0.365	0.450	0.592	1.219	1.329	1.379	0.327	0.392	0.491
wj	**0.131**	**0.173**	**0.241**	**0.206**	**0.303**	**0.429**	**0.145**	**0.202**	**0.296**	**0.220**	**0.322**	**0.480**

Based on the obtained weights for the main criteria, it can be seen that the most important criteria for experts are C4 economic criterion and C2 resistance of the plant / variety. The least important criterion in their opinion is the C1 stem / fruit criterion.

Multiplying the weights of the main criteria with the weights of the sub-criteria, the final values of the weights of the sub-criteria are obtained (Table 2).

**Table 2:** Final values of sub-criteria weights.

C_11_	0.009	0.018	0.043	C_21_	0.035	0.074	0.141	C_31_	0.026	0.051	0.100	C_41_	0.027	0.058	0.124
C_12_	0.014	0.031	0.073	C_22_	0.033	0.071	0.138	C_32_	0.022	0.046	0.095	C_42_	0.027	0.055	0.123
C_13_	0.019	0.046	0.104	C_23_	0.031	0.067	0.136	C_33_	0.021	0.044	0.094	C_43_	0.039	0.083	0.182
C_14_	0.021	0.046	0.105	C_24_	0.024	0.051	0.110	C_34_	0.016	0.033	0.076	C_44_	0.033	0.076	0.172
C_15_	0.016	0.032	0.071	C_25_	0.019	0.041	0.101	C_35_	0.014	0.028	0.068	C45	0.023	0.049	0.114

After determining the weights of the criteria, the experts evaluated the hybrid varieties of sugar beet with assessments in the form of linguistic values (Table 3).

**Table 3:** Initial decision matrix.

Expert 1	C11	C12	C13	C14	C15	C21	C22	C23	…	C43	C44	C45
A1	M	VG	VG	G	MG	MG	MG	G	…	MB	B	M
A2	M	G	G	G	M	G	MG	MG	…	M	M	MB
A3	M	MG	MG	VG	G	G	G	M	…	M	MB	MG
A4	G	VG	MG	G	MG	MG	G	M	…	MB	B	MB
A5	M	MG	MG	MG	MG	MB	MG	M	…	MB	MB	G
A6	M	MG	M	MB	M	M	MG	MG	…	M	MG	M
Expert 2	C11	C12	C13	C14	C15	C16	C17	C21	…	C36	C41	C42
A1	MB	B	VG	VG	M	VG	G	G	…	G	MG	MG
A2	MG	MG	G	G	M	G	G	MG	…	G	G	MG
A3	M	MG	MG	M	M	MG	MG	MG	…	M	M	M
A4	MB	MG	MG	MB	MB	MG	MG	M	…	MB	MB	MB
A5	M	M	M	M	M	M	MB	MB	…	MB	MB	B
A6	MB	M	M	M	M	MG	MB	B	…	MB	B	MG
Expert 3	C11	C12	C13	C14	C15	C16	C17	C21	…	C36	C41	C42
A1	MG	G	VG	G	G	VG	VG	G	…	G	MG	B
A2	G	G	G	G	VG	G	VG	G	…	MG	MG	MB
A3	MG	MG	MG	MG	MG	G	VG	G	…	MG	G	G
A4	MG	M	MB	MB	MB	G	MG	MG	…	M	M	MB
A5	MB	M	MB	MB	MB	MG	M	MG	…	MB	MB	M
A6	MB	MB	MB	MB	B	M	MB	B	…	M	M	M
Expert 4	C11	C12	C13	C14	C15	C16	C17	C21	…	C36	C41	C42
A1	MG	MG	VG	G	MG	G	G	G	…	G	G	G
A2	M	MB	G	G	M	VG	VG	VG	…	VG	G	MG
A3	G	MG	G	VG	M	MG	MG	MG	…	MG	M	M
A4	M	MB	MB	M	MG	G	MG	MG	…	M	MG	M
A5	M	MG	MG	MG	VG	G	M	M	…	MB	MG	MB
A6	M	M	MG	VG	M	G	G	VG	…	G	MB	B
Expert 5	C11	C12	C13	C14	C15	C16	C17	C21	…	C36	C41	C42
A1	MB	M	VG	VG	MG	G	G	G	…	MG	G	M
A2	MG	MG	VG	VG	M	VG	VG	G	…	VG	G	G
A3	B	MB	VG	VG	MG	VG	G	G	…	MB	G	VG
A4	MB	MG	M	M	M	VG	MG	MG	…	MG	MG	MG
A5	G	MG	MG	MG	MG	G	G	VG	…	MG	MG	MG
A6	M	M	MG	MG	MB	MG	G	MG	…	M	M	M

These linguistic values are transformed into fuzzy numbers using the affiliation function. The mean value of the expert opinion is then calculated using the geometric mean. In this way, a common fuzzy decision matrix is formed (Table 4).

**Table 4:** A common fuzzy decision matrix.

CRITERIA	C11	C12	C13	C14	…	C45
A1	3.0 5.0 7.0	4.8 6.4 7.8	9.0 10.0 10.0	7.8 9.4 10.0	…	3.6 5.4 7.2
A2	4.6 6.6 8.4	5.0 7.0 8.6	7.4 9.2 10.0	7.4 9.2 10.0	…	3.8 5.8 7.6
A3	3.6 5.4 7.2	4.2 6.2 8.2	6.2 8.0 9.4	7.0 8.4 9.2	…	5.4 7.2 8.6
A4	3.4 5.4 7.2	4.6 6.4 8.0	3.0 5.0 7.0	3.0 5.0 6.8	…	2.2 4.2 6.2
A5	3.4 5.4 7.2	4.2 6.2 8.2	3.8 5.8 7.8	3.8 5.8 7.8	…	3.2 5.0 6.8
A6	2.2 4.2 6.2	3.0 5.0 7.0	3.4 5.4 7.4	3.8 5.6 7.2	…	2.8 4.6 6.6
max	4.6 6.6 8.4	5.0 7.0 8.6	9.0 10.0 10.0	7.8 9.4 10.0	…	5.4 7.2 8.6

After the common decision matrix is formed, the maximum value for the sub-criteria is calculated and this decision matrix is normalized. The next step of the MABAC method is to multiply the normalized decision matrix by the corresponding weights and add them to those weights. In such a way, an extended decision matrix was obtained. An approximate border area matrix is then determined and these values are subtracted from the extended decision matrix. The next step is to add the appropriate values of the fuzzy numbers and phase the fuzzy numbers. The alternatives are then ranked so that the best alternative is the one with the highest *S*_*i*_ value (Table 5). In this research, the best ranked hybrid variety of sugar beet is A2—Zorica, followed by the A1—NS Ras, while the worst ranked variety is, according to results, A6—Jovana.

**Table 5:** Final ranking order alternatives.

*S*_*1*_	*S*_*2*_	*S*_*3*_	*S*_*i*_	Rank
-3.28	0.12	3.53	0.124	**2**
-3.27	0.14	3.57	0.144	**1**
-3.30	0.08	3.48	0.084	**3**
-3.37	-0.05	3.28	-0.049	**4**
-3.41	-0.13	3.13	-0.131	**5**
-3.41	-0.13	3.11	-0.138	**6**

In order to confirm the obtained results using fuzzy MABAC, the application and calculation of rank orders will be performed using other fuzzy methods.

## 6. Sensitivity analysis and comparison analysis

Sensitivity analysis was three ways. The first way was to examine the results obtained by the fuzzy MABAC method. This was done in such a way that the ranking order obtained by this method was compared with the ranking orders obtained using other fuzzy methods [46]. Another way to conduct sensitivity analysis is to examine how a change in the weight of sub-criteria affects the ranking order of alternatives [47].

**Fig 2:**
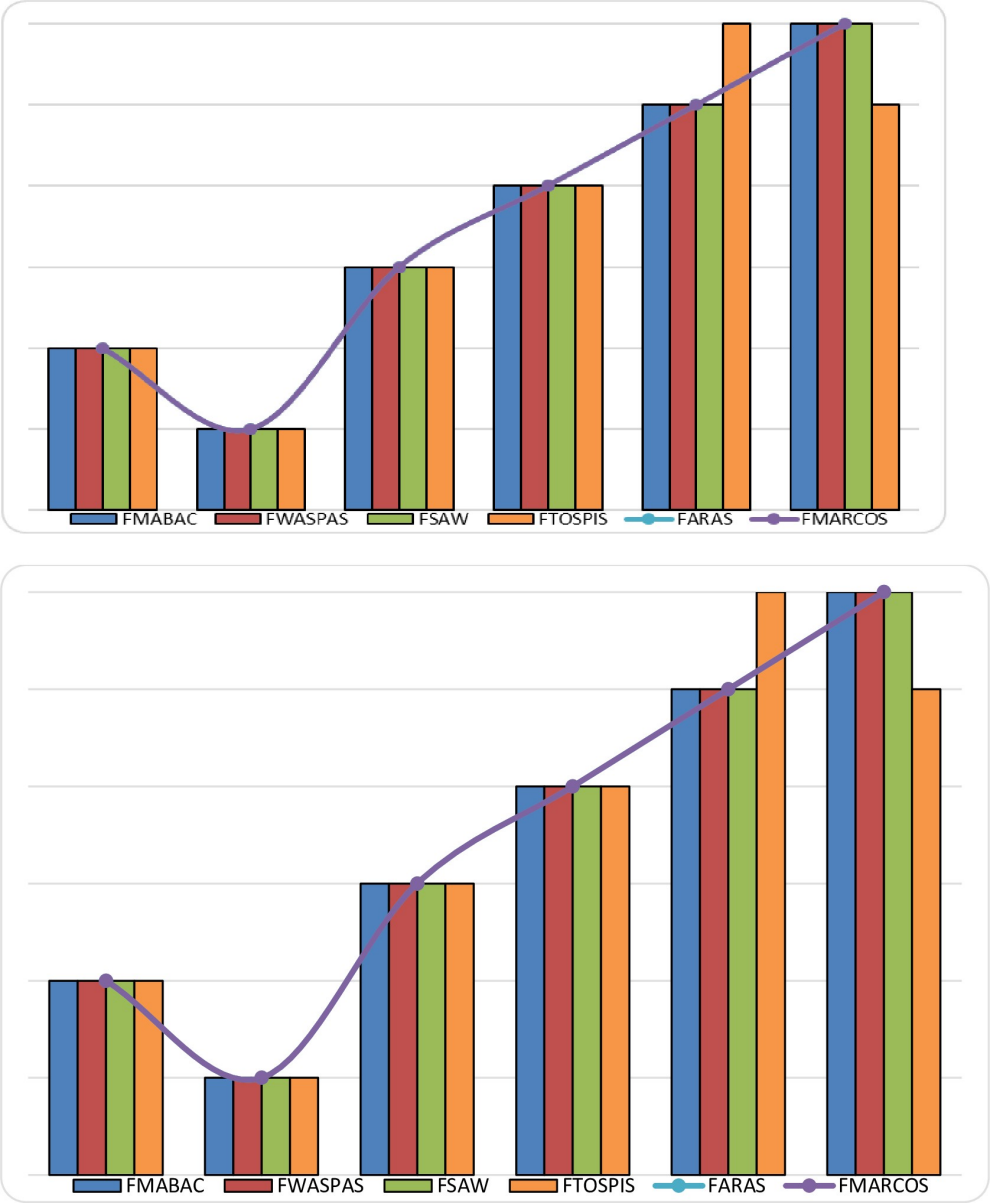
Ranking order obtained using different alternative fuzzy methods.

The ranking of alternatives was performed using the following methods: fuzzy WASPAS (Weighted Aggregated Sum Product Assessment) [31], fuzzy SAW (Simple Additive Weighting technique) [31], fuzzy MARCOS (Measurement Alternatives and Ranking according to the Compromise Solution) [48], fuzzy ARAS (Additive Ratio Assessment) [31], fuzzy TOPSIS (Technique for Order Performance by Similarity to Ideal Solution) [49]. The results obtained show that there is no difference in the ranking of alternatives (Fig 2), so it can be concluded that the fuzzy MABAC method can be used to determine the ranking of alternatives in this case hybrid varieties of rapeseed, because it gives the same result as other fuzzy methods. As evidenced by this sensitivity analysis in these decision-making problems in agriculture, fuzzy logic and various fuzzy methods can be used.

When conducting a second sensitivity analysis, it is necessary to use different scenarios that represent different weights of the sub-criteria. Since there are 20 sub-criteria in the decision-making model, 21 scenarios were used here, and in 20 scenarios one of the criteria was assigned the weight 0.24 while the other criteria were assigned the weight 0.04, in 21 scenarios all sub-criteria were assigned the same weight:” (Table 6). In this way, in the first 20 scenarios, more weight was given to individual criterion than to other criteria, and the impact of this sub-criterion on the ranking of the alternative was observed. The 21^st^ scenario does not give preference to any sub-criteria, but observes all them equally.

**Table 6:** Scenarios in sensitivity analysis.

Sc.	C_11_	C_12_	C_13_	C_14_	C_21_	C_22_	C_23_	C_24_	C_31_	C_32_	C_33_	C_34_	C_41_	C_42_	C_43_	C_44_	C_51_	C_52_	C_53_	C_54_
S1	**0.24**	0.04	0.04	0.04	0.04	0.04	0.04	0.04	0.04	0.04	0.04	0.04	0.04	0.04	0.04	0.04	0.04	0.04	0.04	0.04
S2	0.04	**0.24**	0.04	0.04	0.04	0.04	0.04	0.04	0.04	0.04	0.04	0.04	0.04	0.04	0.04	0.04	0.04	0.04	0.04	0.04
S3	0.04	0.04	**0.24**	0.04	0.04	0.04	0.04	0.04	0.04	0.04	0.04	0.04	0.04	0.04	0.04	0.04	0.04	0.04	0.04	0.04
. . .	. . .	. . .	. . .	. . .	. . .	. . .	. . .	. . .	. . .	. . .	. . .	. . .	. . .	. . .	. . .	. . .	. . .	. . .	. . .	. . .
S18	0.04	0.04	0.04	0.04	0.04	0.04	0.04	0.04	0.04	0.04	0.04	0.04	0.04	0.04	0.04	0.04	0.04	**0.24**	0.04	0.04
S19	0.04	0.04	0.04	0.04	0.04	0.04	0.04	0.04	0.04	0.04	0.04	0.04	0.04	0.04	0.04	0.04	0.04	0.04	**0.24**	0.04
S20	0.04	0.04	0.04	0.04	0.04	0.04	0.04	0.04	0.04	0.04	0.04	0.04	0.04	0.04	0.04	0.04	0.04	0.04	0.04	**0.24**
S21	**0.05**	**0.05**	**0.05**	**0.05**	**0.05**	**0.05**	**0.05**	**0.05**	**0.05**	**0.05**	**0.05**	**0.05**	**0.05**	**0.05**	**0.05**	**0.05**	**0.05**	**0.05**	**0.05**	**0.05**

**Fig 3:**
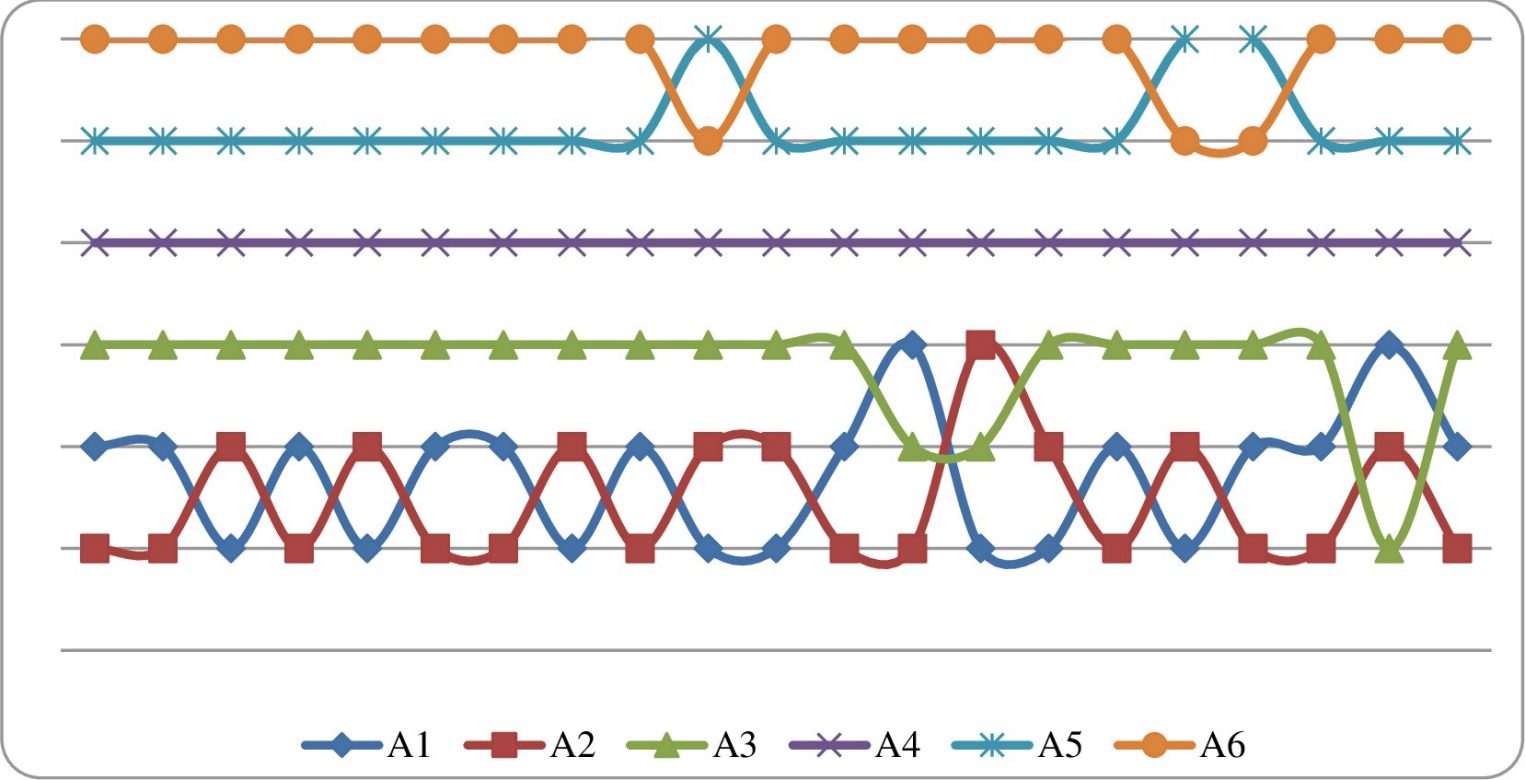
Ranking of the alternative by applying sensitivity analysis.

The results obtained by applying different scenarios show that as many as 3 hybrid varieties of rapeseed occupy the first place by applying a certain scenario. Alternative A2 takes first place in the ranking in 12 scenarios, alternative A1 takes first place in 8 scenarios while alternative A3 takes first place once in the application of scenario 20. In this way you can see which scenarios are sensitive to each alternative. Take for example alternative A2, it is sensitive to 8 scenarios because then it takes second or third place. So, we can say that this alternative is particularly sensitive to the application of scenario 14 when it took third place in the ranking. Alternative A2 has a poorer ability to store fruit compared to alternatives A1 and A3. The situation is similar in the other 8 scenarios where alternatives A1 and A3 have a better ranking compared to alternatives A2. Alternative A4 did not show any sensitivity to changes in the weights of the sub-criteria as it ranked fourth in all scenarios used. Alternative A5 ranked fifth in 18 scenarios while Alternative A6 ranked fifth in 3 scenarios. Alternatives A5 and A6 showed the worst indicators with this analysis. In order for any alternative to be better, it must improve the characteristics according to a certain sub-criterion that influenced the lower ranking. To conclude, it is necessary to improve that variety in terms of that sub-criterion (Fig 3).

The Bonferroni aggregator was applied to aggregate expert’s preferences in calculating the values of weight coefficients in the fuzzy PIPRECIA model. When calculating the initial values of the fuzzy weights of the criteria in the Bonferroni function, the parameter values were adopted *p = q =* 1. Many studies [35, 50, 51] indicate the fact that changes in the values of the parameters *p* and *q* can lead to changes in the aggregate values, and thus in the final rang of alternatives. Therefore, the following section presents an analysis of the impact of changes in the parameters *p* and *q* on the change in the values of the weight coefficients of the criteria and values in the aggregate decision matrix.

**Fig 4:**
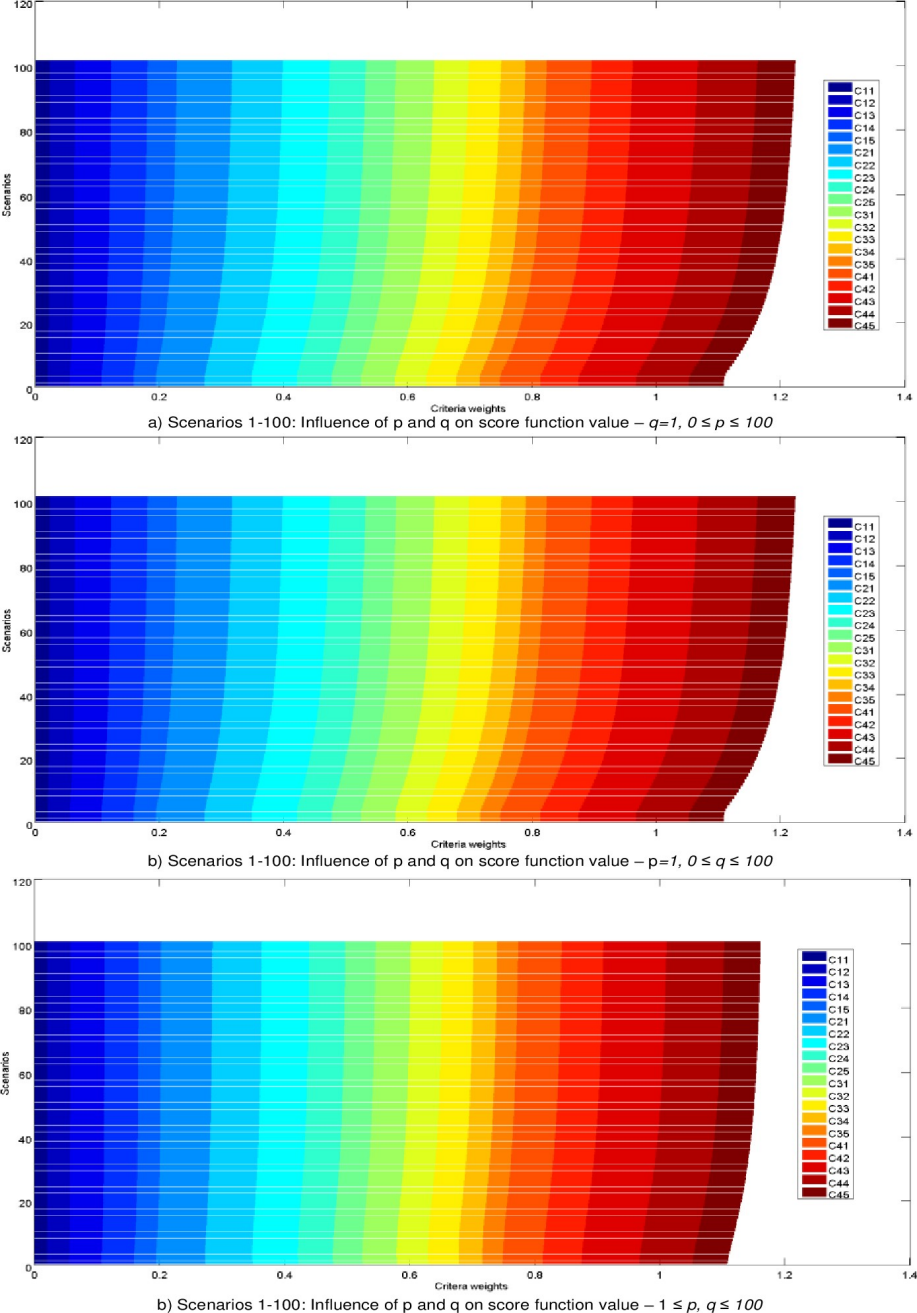
Influence of parameters *p* and *q* on the change of values of weight coefficients of criteria.

In the next part, the influence of changing the parameters *p* and *q* through 300 scenarios is simulated and 300 new vectors of weight coefficients are formed. Thus, through the first 100 scenarios, the change of the parameter value was simulated 0≤*p*≤100, while value of parameter *q* staying same, ie. *q = 1*. In the next 100 scenarios the change of the parameter value was simulated 0≤*q*≤100, while value of parameter *p* is unchanged ie. *p = 1*. Fig 4A and 4B. In the last 100 scenarios changing of both parameters in range 1≤*p*,*q*≤100, is simulated (Fig 4C).

**Fig 5:**
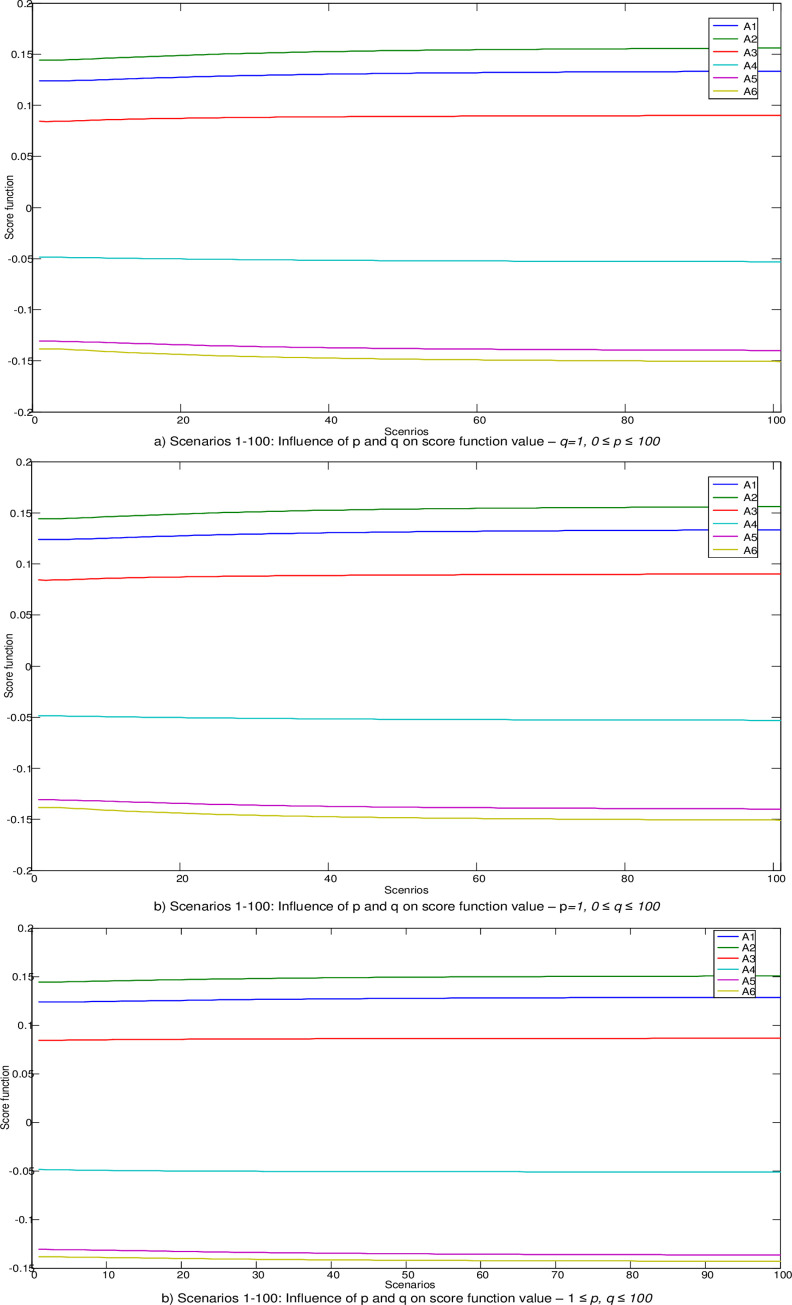
Influence of parameters *p* and *q* on the change of the values of the criterion functions in the fuzzy MABAC.

After determining the new vectors of weight coefficients, their influence on the change of the values of the criterion functions in the fuzzy MABAC model was analyzed (Fig 5).

By analyzing the results from Fig 5, we notice that for changes in the values of parameters through all 300 do not lead to a change in the rank of alternatives and that the initial rank A2> A1> A3> A4> A5> A6 is confirmed. It has been confirmed that changes in the values of the parameters p, q lead to a change in the values of the criterion functions, however these changes are not large enough to cause changes in the initial rank. Also, the presented results showing that the initial solution is credible and robust despite drastic changes in the input parameters.

## 7. Conclusion

Each sowing is an investment for farmers. They have to invest certain funds for land preparation, seed procurement and sowing, and the return on that investment is expected after the harvest. Therefore, when choosing a variety, it is necessary to choose the one that will bring them the best results. In order to be able to choose a certain variety at all, it is necessary to consider the characteristics of that variety. The characteristics are considered on the basis of several criteria, and MCDM methods are used to make a decision on the source of the variety.

This paper, on the example of hybrid varieties of rapeseed developed at the Institute of Field and Vegetable Crops in Novi Sad, ranked these varieties using a novel integrated fuzzy MCDM model. Fuzzy logic enables the application of linguistic values ​​or evaluations for assessment as close as possible to human thinking. In order to apply fuzzy logic, it was necessary to create a decision model for this problem. Assistance of five anonymous experts was used to create a decision-making model on the basis of which individual varieties were evaluated. The fuzzy MABAC method was used to rank these alternatives.

The results obtained using the fuzzy MABAC method showed that based on the weights determined by five experts, the hybrid rapeseed variety A2—Zorica has the best results, followed by the A1 NS Ras variety, while the A5—Zlatna variety has the worst results. These results were confirmed using other fuzzy methods. Sensitivity analysis showed that variety A6—Jovana has the worst results, taking the last place in the application of 18 scenarios.

The main limitation of this research is the analysis of 6 hybrids produced at the Institute of Field and Vegetable Crops in Novi Sad. Taking a larger number of hybrids implies the problem of evaluating them. It is of huge importance to give equal conditions to all hybrids and monitor them throughout the year, and only then, based on that monitoring, it is necessary to give an assessment for each hybrid. As this number increases, the process of selecting the best hybrid becomes more complicated.

The conducted research provided good settings on how to solve the problem of decision-making in agriculture regarding the choice of variety for sowing. In addition, the obtained results showed advantages and disadvantages of certain hybrid varieties of rapeseed. These results will help farmers choose the variety of rapeseed that will give them the best results.

In future research, it would be suitable to apply this methodology in order to perform the analysis of other hybrids and thus select the best basis for the development of new types of hybrids. Only on the best hybrids it is possible to create even better samples and thus increase the production of rapeseed by farmers.
